# Relationship of Blood Group with Level of Cooperation of Pediatric Dental Patients

**DOI:** 10.1155/2022/7147740

**Published:** 2022-06-06

**Authors:** Alireza Heidari, Marzieh Salehi Shahrabi, Elnaz Askari Anaraki

**Affiliations:** Department of Pediatric Dentistry, School of Dentistry, Tehran University of Medical Sciences, Tehran 14399-55991, Iran

## Abstract

Acquaintance with the behavior of children in dental office setting is highly important in treatment success. People with different blood groups often have different behaviors. Thus, the blood group may aid in prediction of behavior of pediatric dental patients. This study is aimed at assessing the relationship of the blood group with level of cooperation of pediatric dental patients. This cross-sectional study was conducted on 130 children between 4 and 6 years of age. The blood group of children was recorded according to their identification card or by collecting an intraoral blood sample during pulpotomy and using the respective kit. To assess the level of cooperation of children, their behavior was videotaped during the procedure, and the videos were assessed by two pedodontists. The behavior of children was scored according to the Venham scale. Data were analyzed by SPSS 26 and Chi-square, Fisher's exact test, and Mann–Whitney *U* test. The blood group was A in 35.7%, B in 5.8%, AB in 3.2%, and O in 53.5%. Children with blood group O had maximum cooperation (52.6) while those with blood group B had minimum cooperation. Pairwise comparisons of the groups regarding the Venham scale revealed significant differences between blood groups A and B (*P* = 0.0001) and also B and O (*P* = 0.005). Pairwise comparisons of the groups regarding the level of cooperation also revealed significant differences between blood groups A and B (*P* = 0.0001) and B and O (*P* = 0.019). Blood group B may be correlated with certain behavioral traits such as dental fear and anxiety and the resultant poor cooperation.

## 1. Introduction

It is important to raise awareness regarding the significance of primary and permanent teeth and their role in growth and development of children, especially among the parents. The basis of pediatric dentistry is behavioral control of children and establishing an efficient communication between pediatric patients and their clinician in all steps of treatment [1]. This is a prerequisite for provision of high-quality care to pediatric dental patients. Prediction of the behavior and reactions of children to diagnostic and therapeutic procedures is a common challenge for dental clinicians. According to Hetherington et al. [2] and Brill [3], prediction of the behavior of children in different situations is highly important in success of dental treatment. Depending on their personality type, children may show different behaviors and variable levels of cooperation in dental office. Proper assessment of the behavior of children can help dental clinicians in arrangement of dental visits and efficient treatment planning [4, 5]. Considering the significance of familiarity with the behavior of children and knowledge about their behavioral management in dental office setting, a large body of research in pediatric dentistry focuses on this topic [6–10]. Dental clinicians visiting pediatric dental patients are interested in knowing novel techniques and criteria to predict the behavior and reactions of pediatric dental patients [4, 11, 12].

The behavior of children in dental office setting is influenced by a number of genetic and environmental factors [2, 3, 13–18]. Although human characteristics are dictated by the genes, some characteristics such as intelligence, height, and excitability cannot be linked to only one gene. The blood group is among the most important genetic properties that is believed to be implicated in many personal characteristics. At present, the blood group is used as an important, noninvasive, and low-cost diagnostic tool to acquire information about different disease conditions. Numerous studies have evaluated the relationship of the blood group with different diseases. However, most of such studies focused on physical, and not psychological, conditions, and the relationship of the blood group with psychological disorders and antisocial behaviors has been less commonly addressed [19–21].

In dental science, the correlation of the blood group with some oral and dental conditions has been investigated. For instance, the correlation of the blood group with periodontal disease, dental caries, and malocclusion has been previously studied [22–27]. However, the relationship of the blood group with the behavior of pediatric dental patients and their level of cooperation has not been previously addressed. Considering the significance of cooperation of children and their behavioral control in dental office setting, this study is aimed at assessing the possible relationship of the blood group with level of cooperation of pediatric dental patients.

## 2. Materials and Methods

This descriptive cross-sectional study was conducted at the Pediatric Dentistry Department of School of Dentistry, Tehran University of Medical Sciences, on 130 children between 4 and 6 years of age, who were selected by convenience sampling in 2020. All parents signed informed consent forms for participation of their children in the study, and the study protocol was approved by the ethics committee of Tehran University of Medical Sciences (IR.TUMS.DENTISTRY.REC.1399.045).

The inclusion criteria were absence of systemic medical conditions (ASA class I or II); no history of hospitalization; no history of previous dental treatment; no history of psychopathological, psychological, or anxiety disorders; having at least one primary mandibular molar requiring pulp therapy; and willingness and consent of the parents for participation in the study.

The exclusion criteria were unwillingness for participation in the study and dental emergencies such as traumatic dental injuries or dental pain.

### 2.1. Methodology

The blood group of children was first recorded according to their identification card. For children who did not have their identification card with them, their blood group was determined by intraoral blood collection during the pulpotomy treatment using a blood type kit (Sinagen, Iran).

All dental treatments were performed by a postgraduate student of pediatric dentistry who was not aware of the blood group of children during dental procedures. The behavior of children during the procedures was videotaped to classify their level of cooperation and anxiety during the procedure using the Venham scale [28]. The video camera was fixed on a tripod in front of dental unit such that the head, hands, and feet of the patient were completely visible within the field of view.

#### 2.1.1. First Treatment Session

The children entered the operatory with their mothers and were requested to sit on the dental chair. In this session, only a dental mirror and a dental explorer were used for oral and dental examination, and then, dental prophylaxis was performed with a rubber cup and handpiece. In this session, the tell-show-do technique was used to communicate with the children and control their behavior.

#### 2.1.2. Second Treatment Session

The second treatment session was scheduled 1-2 weeks after the first session. The children entered the operatory alone. The attending postgraduate student of pediatric dentistry helped the children to sit on dental chair. The camera was turned on by dental assistant before sitting the child on dental chair. The camera was positioned such that it did not attract any attention.

Topical anesthetic agent (20% benzocaine gel; Master-Dent) was applied on the injection site, and an inferior alveolar nerve block injection was then administered (1 mL of 2% lidocaine with 1 : 100,000 epinephrine) prior to pulp therapy of primary mandibular molar tooth. Pulp therapy was then performed. The mean duration of procedure was recorded, and patients whose procedures lasted longer than 30-45 minutes for any reason were excluded.

The videos were independently viewed by two faculty member pedodontists, and the behavior of the children was evaluated and scored at two phases of anesthetic injection and pulp therapy using the Venham scale [28]. Children who were highly uncooperative in the first treatment session and required sedation or general anesthesia were categorized as uncooperative children. Data were analyzed using SPSS version 26 and Chi-square, Fisher's exact test, and Mann–Whitney *U* test.

## 3. Results

Data of 130 children between 4 and 6 years were collected; of which, 75 (58.1%) were females, and 54 (41.9%) were males. In terms of frequency of the blood groups, 35.7% had blood group A, 8.5% had blood group B, 2.3% had blood group AB, and 53.5% had blood group O; 92.2% were Rh positive, and 7.8% were Rh negative.

Regarding the level of cooperation of children, 25.8% were uncooperative (low cooperation or no cooperation), and 74.2% were cooperative. According to the Venham rating scale, 50% of children were assigned to subgroup 0, 24.2% were assigned to subgroup 1, 18% were assigned to subgroup 2, 0.8% were assigned to subgroup 3, 7% were assigned to subgroup 4, and 0% were assigned to subgroup 5. Of all, 73.3% of girls and 75.5% of boys were cooperative, and no significant correlation was found between gender and level of cooperation of children (*P* = 0.78). The mean age of cooperative children was 5.31 ± 0.79 years, and the mean age of uncooperative children was 5.16 ± 1.03 years (*P* = 0.37).

**Figure 1:**
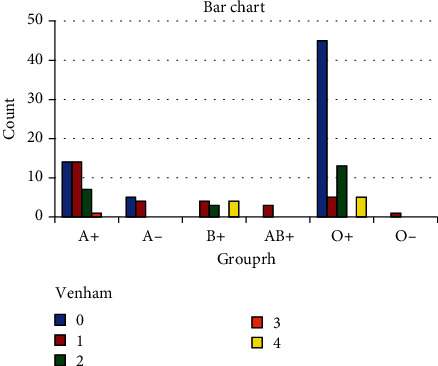
Relationship of the blood group with the Venham scale.

Data analysis revealed that the Venham scale 0 had the highest frequency in blood group O+ (70.3%) (Table 1 and Figure 1). The level of cooperation was the highest in children with blood group O+ (52.6, Table 2). The lowest level of cooperation (Venham scale 4) had the highest frequency in blood group B (44.4%). Also, children with blood group B had the lowest level of cooperation (21.2%, Table 2).

Pairwise comparisons of the blood groups regarding the Venham scale by the Mann–Whitney test showed significant differences between blood groups A and B (*P* = 0.0001) and B and O (*P* = 0.005). Pairwise comparisons of the blood groups regarding the level of cooperation by the Mann–Whitney test showed significant differences between blood groups A and B (*P* = 0.0001) and also B and O (*P* = 0.019) (Table 3).

## 4. Discussion

This study assessed the possibility of prediction of the behavior and level of cooperation of pediatric dental patients based on their blood group. Obviously, such prediction after the first treatment session can greatly help in treatment planning and scheduling of the next appointments. Primary assessment of this possible correlation revealed higher frequency of blood group B among uncooperative children and higher frequency of blood groups A, O, and AB among cooperative children. This difference was significant. Also, all Rh- children and the majority of Rh+ children were cooperative. Search of the literature by the authors revealed that the present study is probably the only available study on the correlation of the blood group and level of cooperation of pediatric dental patients. Thus, the obtained results cannot be compared with those of other studies. Since many investigations are available on the correlation of behavioral characteristics with the blood group, the results of such studies are discussed here.

The personal and behavior of different individuals are widely variable, and people show different behaviors in different situations and under different circumstances. The blood group may determine the perception of excitements, motivations, and performance of different individuals in different situations. Evidence shows differences in behavior and personally of individuals with different blood groups [19–21].

The present results indicated that according to the Venham scale, 25.8% of children were uncooperative, and 74.2% were cooperative. In a study by Colares and Richman [28], the behavior of children was completely positive in 1%, positive in 59%, negative in 28%, and completely negative in 12%. In this study also, the overall percentage of children with positive behavior was higher than the percentage of children with negative behavior, which was in line with the present results. However, small differences existed in the absolute values, which can be due to racial, cultural, and demographic parameters and the type of behavior rating scale used. Sasaki et al. [29] in Japan reported that people with blood group O had higher likelihood of being blood donors than other groups. They added that people with blood group O had a friendlier behavior than others. In the present study, children with blood group O showed a more positive behavior according to the Venham scale. It may be stated that more friendly behavior of this group results in their better cooperation in dental office setting. Also, Tehrani [30] reported that people with blood group AB+ were less extroverted and more rational and adaptable, those with blood group A+ were more impulsive and cooperativeness, and those with blood groups O+ and A+ were more sociable than those with blood group AB+. Those with blood group B+ were mostly unstable and erratic, strong, and decisive, and those with blood group O+ were more inspirable. In the present study, children with blood groups O, A, and AB had a more positive behavior according to Venham scale, and those with blood group B were more uncooperative. It may be stated that more friendly nature of children with blood groups O and A results in their better cooperation, while emotional instability in blood group B results in lower level of cooperation. Moreover, inspiration in children with blood group O may lead to their higher level of cooperation in dental office. Another study on the correlation of personality traits and blood group reported that patients with blood group B had higher level of psychopathological disorders, and those with blood group O were more extroverted. Those with blood group A were more sociable than others, and individuals with blood group AB showed higher level of sacrifice and selflessness [31]. Accordingly, individuals with blood group B had higher level of negative emotions and also showed lower level of cooperation in our study, which may be explained by their negative emotions and thoughts towards dentistry. Nahida and Chatterjee [32] in 2016 evaluated the correlation of the blood groups and personality traits by using the revised version of the Eysenck Personality Questionnaire introduced by Eysenck in 1975 [33]. The Chi-square test showed no significant correlation between the blood group and personality while in the present study, a significant correlation was noted between the behavior of children in dental office and their blood group. This controversy in the results of the two studies can be due to different behavior assessment scales used and different age groups of children. Asgari [34] indicated that the blood group affected the level of cooperation and teamwork, and individuals with blood group A were more interested in teamwork followed by those with blood groups O, AB, and B. Their results were somehow in line with the present findings since children with blood group B were less cooperative in the present study, which may be due to their poor teamwork, and those with blood groups A and O showed higher level of cooperation according to Venham scale.

Some studies focused on the correlation of the blood group with some psychological disorders such as depression [19, 35, 36]. Pisk et al. [19] in 2019 evaluated the correlation of the blood group with psychological disorders and found that a significant percentage of those with psychological disorders had blood group AB, compared with healthy controls. Accordingly, the prevalence of psychological disorders was approximately 3 times higher in individuals with blood group AB, and no correlation was noted between the ABO blood groups and other parameters (gender, psychological heredity, and suicide). Their results confirmed the correlation of psychological disorders and ABO blood groups. Difference between their results and present findings may be due to assessment of psychological disorders in the study by Pisk et al. and behavior in dental office in the present study. Qadir and Hanif [35] in 2018 evaluated the correlation of the blood group with depression and concluded that rate of depression was higher in B+ males and females and lower in AB- males and females. This finding can explain the high percentage of B+ children in the uncooperative group.

The origin of fear in 4 to 6 years old is multifactorial, and one limitation of this study was that no means were used to evaluate the children's general fears and possible experiences in dental or health care services in general. The children's behavior can be due to fear induced by their friends or family or treatment avoidance since it is a new experience. Other limitations of this study included the parents' reluctance to make a second appointment and the loss of the blood sample during preparation and transfer to the kit.

As mentioned earlier, no similar study is available to compare our results with. The present study showed a possible correlation between blood group and level of cooperation of pediatric dental patients. Thus, the behavior of children may be predicted based on their blood group. However, this study was a single-center study and had a cross-sectional design and a small sample size. Further studies with a larger sample size are recommended on children from different populations and socioeconomic classes.

## Figures and Tables

**Table 1:** Relationship of the blood group with the Venham scale.

Venham	0	1	2	3	4	5	Total
Blood group							
A+							
Number	14	14	7	1	0	0	36
Percentage	21.9	45.2	30.4	100	0.0	0.0	28.1%
A-							
Number	5	4	0	0	0	0	9
Percentage	7.8	12.9	0.0	0.0	0.0	0.0	7.0%
B+							
Number	0	4	3	0	4	0	11
Percentage	0.0	12.9	13.0	0.0	44.4	0.0	8.6%
AB+							
Number	0	3	0	0	0	0	3
Percentage	0.0	9.7	0.0	0.0	0/0	0.0	2.3%
O+							
Number	45	5	13	0	0	0	68
Percentage	70.3	16.1	56.5	0.0	0.0	0.0	53.1%
O-							
Number	0	1	0	0	1	0	1
Percentage	0.0	3.2	0.0	0.0	0.3	0.0	0.8

**Table 2:** Comparison of the blood groups regarding the level of cooperation.

Blood group	Uncooperative (poor cooperation to highly uncooperative)	Cooperative and highly cooperative
A+		
Number	8	28
Percentage	24.2	29.5
A-		
Number	0	9
Percentage	0.0	9.5
B+		
Number	7	4
Percentage	21.2	4.2
AB+		
Number	0	3
Percentage	0.0	3.2
O+		
Number	18	50
Percentage	54.4	52.6
O-		
Number	0	1
Percentage	0.0	1.1

**Table 3:** Pairwise comparisons of the blood groups regarding level of cooperation.

Blood group	Level of cooperation	*P* value
A	45	
B	11	0/005
AB	3	0/429
O	69	0/365
B	11	
AB	3	0.192
O	69	0/019
AB	3	
O	69	0/568

## Data Availability

The data used to support the findings of this study were supplied by corresponding author under license, and data will be available on request. Requests for access to these data should be made to corresponding author.
